# A Modified Reading the Mind in the Eyes Test Predicts Behavioral Variant Frontotemporal Dementia Better Than Executive Function Tests

**DOI:** 10.3389/fnagi.2018.00011

**Published:** 2018-01-30

**Authors:** Matthias L. Schroeter, Sarah Pawelke, Sandrine Bisenius, Jana Kynast, Katharina Schuemberg, Maryna Polyakova, Sarah Anderl-Straub, Adrian Danek, Klaus Fassbender, Holger Jahn, Frank Jessen, Johannes Kornhuber, Martin Lauer, Johannes Prudlo, Anja Schneider, Ingo Uttner, Angelika Thöne-Otto, Markus Otto, Janine Diehl-Schmid

**Affiliations:** ^1^Leipzig & Clinic for Cognitive Neurology, University Hospital Leipzig, Max Planck Institute for Human Cognitive and Brain Sciences, Leipzig, Germany; ^2^Department of Neurology, University of Ulm, Ulm, Germany; ^3^Department of Neurology, Ludwig Maximilian University of Munich, Munich, Germany; ^4^Department of Neurology, Saarland University, Homburg, Germany; ^5^Department of Psychiatry and Psychotherapy, University Medical Center Hamburg-Eppendorf, Hamburg, Germany; ^6^Department of Psychiatry and Psychotherapy, University of Bonn, The German Center for Neurodegenerative Diseases, Bonn, Germany; ^7^Department of Psychiatry and Psychotherapy, Friedrich-Alexander University Erlangen-Nürnberg, Erlangen, Germany; ^8^Department of Psychiatry, Psychosomatics and Psychotherapy, University of Würzburg, Würzburg, Germany; ^9^Department of Neurology, University of Rostock, Rostock, Germany; ^10^Department of Psychiatry and Psychotherapy, University of Göttingen, Göttingen, Germany; ^11^Department of Psychiatry and Psychotherapy, Technical University of Munich, Munich, Germany

**Keywords:** behavioral variant frontotemporal dementia, diagnostic criteria, executive function, social cognition, theory of mind

## Abstract

Behavioral variant frontotemporal dementia (bvFTD) is characterized by deep alterations in behavior and personality. Although revised diagnostic criteria agree for executive dysfunction as most characteristic, impairments in social cognition are also suggested. The study aimed at identifying those neuropsychological and behavioral parameters best discriminating between bvFTD and healthy controls. Eighty six patients were diagnosed with possible or probable bvFTD according to Rascovsky et al. (2011) and compared with 43 healthy age-matched controls. Neuropsychological performance was assessed with a modified Reading the Mind in the Eyes Test (RMET), Stroop task, Trail Making Test (TMT), Hamasch-Five-Point Test (H5PT), and semantic and phonemic verbal fluency tasks. Behavior was assessed with the Apathy Evaluation Scale, Frontal Systems Behavioral Scale, and Bayer Activities of Daily Living Scale. Each test’s discriminatory power was investigated by Receiver Operating Characteristic curves calculating the area under the curve (AUC). bvFTD patients performed significantly worse than healthy controls in all neuropsychological tests. Discriminatory power (AUC) was highest in behavioral questionnaires, high in verbal fluency tasks and the RMET, and lower in executive function tests such as the Stroop task, TMT and H5PT. As fluency tasks depend on several cognitive functions, not only executive functions, results suggest that the RMET discriminated better between bvFTD and control subjects than other executive tests. Social cognition should be incorporated into diagnostic criteria for bvFTD in the future, such as in the International Classification of Diseases (ICD)-11, as already suggested in the Diagnostic and Statistical Manual for Mental Disorders (DSM)-5.

## Introduction

Behavioral variant frontotemporal dementia, the most frequent subtype of FTLD, is characterized by deep alterations in behavior and personality (Neary et al., 1998). By focusing on clinical symptoms in histopathologically confirmed cases, an international consortium revised the diagnostic criteria for bvFTD (Piguet et al., 2011; Rascovsky et al., 2011). Here, ‘possible’ bvFTD is defined by at least three of six clinically discriminating features: disinhibition, apathy/inertia, loss of sympathy/empathy, perseverative/stereotyped/compulsive/ritualistic behavior, hype- rorality/dietary changes and, neuropsychologically, deficits in executive functioning. Executive functions, also called executive or cognitive control, represent a wide variety of higher order cognitive processes enabling flexible modification of thought and behavior to environmental changes, although the concept is, at least partly, controversially discussed in the literature. Executive abilities are essential for coping with changing demands of everyday life (Miller and Cohen, 2001). Beyond clinical criteria, biomarkers were additionally included into Rascovsky et al.’s (2011) disease definition to increase diagnostic validity. ‘Probable’ bvFTD requires specific (frontotemporal) neuroimaging findings, whereas bvFTD ‘with definite FTLD’ has to be supported by histopathological confirmation or a pathogenic mutation. The new revised criteria have a much higher sensitivity in comparison to the earlier ones (Neary et al., 1998).

Remarkably, the new DSM-5, published in 2013, has included a prominent decline in social cognition in addition to executive dysfunction to bvFTD’s criteria, here called frontotemporal neurocognitive disorder (American Psychiatric Association, 2013). In analogy to Rascovsky et al. (2011), three behavioral symptoms have to be fulfilled, and validity is increased by neuroimaging and/or causative genetic mutations. Social cognition includes cognitive processes that are important for social interactions (Moskowitz, 2005). It enables recognizing, manipulating, and behaving with respect to socially relevant information (Poletti et al., 2012). Here, social signals have to be perceived and connected to motivation, emotion, and adaptive behavior. One central concept of social cognition is ToM or ‘mentalizing’ referring to the ability to attribute mental states to self and others and to describe, explain and predict behavior on the basis of such mental states (Frith and Frith, 2003; Amodio and Frith, 2006; Poletti et al., 2012). Two components of ToM have been suggested recently, a cognitive component focussed on processing other people’s beliefs and intentions, and an affective component focalized on processing others’ emotions and feelings (Poletti et al., 2012). Several studies have shown specific deficits in social cognition/ToM in bvFTD (Gregory et al., 2002; Rankin et al., 2006; Adenzato et al., 2010; Henry et al., 2014; Schroeter et al., 2014) even predicting its development (Pardini et al., 2013). Whereas performance in ToM tasks does not correlate with executive functioning in early bvFTD, ToM ability and executive functions become strongly related in advanced disease (Seelaar et al., 2011).

In sum, there is consensus in the literature that patients with bvFTD show profound changes in personality/behavior and that bvFTD affects executive functions (Rascovsky et al., 2011; American Psychiatric Association, 2013). Impairments in social cognition are regarded as a neuropsychological and diagnostic hallmark by some authors (American Psychiatric Association, 2013), whereas others regard it as less relevant (Rascovsky et al., 2011) or an epiphenomenon of executive dysfunction (Le Bouc et al., 2012; Schroeter et al., 2014).

To investigate the relevance of social cognition in diagnosing bvFTD clinically, we examined the discriminatory diagnostic power for several neuropsychological tests in a large sample of bvFTD patients compared with age-matched healthy subjects from the multi-centric FTLD consortium’s study Germany (Otto et al., 2011). We applied the new diagnostic criteria for bvFTD (Rascovsky et al., 2011). Tests assessed executive functions, social cognition, and behavioral parameters. Recently, Miyake et al. (2000) have suggested that executive control comprises three core processes, namely working memory, task switching and inhibitory control. To cover these aspects we used different tests for fluency, divided attention and inhibition of overlearned responses. Based on previous studies we hypothesized that executive function tests, in particular verbal fluency tests, are good predictors for bvFTD (Pachana et al., 1996; Galante et al., 1999; Gregory, 1999; Pasquier et al., 1999; Perry and Hodges, 2000).

Recent investigations emphasized a dominant role of social cognition in the early diagnosis of bvFTD and suggested that these tests might constitute a better diagnostic predictor than traditional executive tests (Gregory et al., 1999, 2002; Hodges, 2007; Torralva et al., 2009; Adenzato et al., 2010; Poletti et al., 2012; Adenzato and Poletti, 2013; Pardini et al., 2013; Bora et al., 2015). Accordingly, we further hypothesized that bvFTD patients show a decline in social cognition as compared to healthy controls and that social cognition tests have more diagnostic power in the identification of bvFTD than traditional tests of executive functions. Here, we applied a test covering cognitive and affective components of ToM. Finally, we hypothesized that informant-report questionnaires are more sensitive than self-report questionnaires in detecting behavioral changes in bvFTD due to unawareness in these patients (Schroeter et al., 2014).

## Materials and Methods

### Patients

Data were provided by the multi-centric FTLD consortium’s study Germany (Otto et al., 2011^1^). The cohort included 86 patients diagnosed with possible and probable bvFTD according to Rascovsky et al. (2011) and 43 healthy age-matched control subjects. Note that, accordingly, deficits in social cognition were not included in the diagnostic criteria thereby preventing a circular study design. Patients and control subjects were age-matched on a two to one basis within the range of ±4 years. The standardized protocol included a range of clinical, neuropsychological, brain imaging, and cerebrospinal fluid biomarker assessments. The study was approved by the ethics committees of all Universities contributing patients and controls, and was in accordance with the latest version of the Declaration of Helsinki (ethics committee Leipzig ID 137-11-18042011). Each participant provided written informed consent.

### Neuropsychological and Behavioral Tests

In the following, clinical rating scales, questionnaires, and neuropsychological tests are described. The CDR evaluates the severity and stage of dementia (Morris, 1993). Because CDR was originally designed for Alzheimer’s disease, an FTLD-CDR was additionally included considering also behavior/personality and language (Knopman et al., 2008; range 0–18/24). Education was operationalized with the ISCED into seven levels (Organization for Economic Co-Operation and Development, 1999).

Questionnaires were conducted based on informant- and self-report. The AES quantifies and characterizes apathy (range 0–54; Marin et al., 1991). The BADL rates global difficulties in everyday life activities (range 1–10; Hindmarch et al., 1998). The modified FrSBe measures behavior associated with frontal lobe damage (subscales apathy, disinhibition, executive dysfunction; range 24–120; Grace and Malloy, 2001).

Neuropsychological tests assessed executive functions and social cognition. The *Stroop color-word interference task* measures interference resolution and response inhibition (percentage of correct answers in 45 s; MacLeod, 1991). Subjects are requested to name the color of a word with an incongruent meaning. By doing this they have to inhibit an overlearned response (reading) in favor of a novel response (color naming) (Schroeter et al., 2002, 2004). The TMT consists of two parts. Part A requires subjects to connect numbers, whereas in Part B an alternating sequence between numbers and letters has to be drawn. Thus, Part B assesses mental flexibility and divided attention (Lezak et al., 2012). The B/A ratio for completion time as used in our study measures executive function (Arbuthnott and Frank, 2000). A further aspect of executive functions is spontaneous divergent thinking. The H5PT assesses figural divergent thinking or figural fluency (Haid et al., 2002). Subjects have to connect five point boxes in varying ways to create as many different patterns as possible (percentage of correct patterns within three min). Executive functions can also be evaluated by verbal fluency tests. In these tests, subjects have to produce as many words as possible that begin with a specific letter (*phonemic fluency*; s-words) or belonging to a specific category (*semantic fluency*; animals) (Crawford and Henry, 2005) (correct answers within one min). Besides assessing spontaneous divergent thinking, the test also provides information about speech ability and, in the case of the categorical task, about semantic memory capabilities (Morris et al., 1989; Lezak et al., 2012). Finally, a modified RMET was applied to measure aspects of social cognition. Here, 18 photographs of the eye-region of human faces are presented to the subject (Baron-Cohen et al., 2001). Additionally, six photographs of the human eye-region contained basic emotions resulting in 24 items in total. Accordingly, the test covered both cognitive and affective components of ToM. As shown in a recent comprehensive meta-analysis, bvFTD patients showed substantial impairment on ToM and emotion recognition tasks compared with healthy controls, without significant differences between both measures, justifying its combination (Henry et al., 2014). Subanalyses were not performed due to low respective item numbers. In the task, the subject is required to choose among four adjectives describing what the individual in the photography is thinking or feeling (number of correct answers). The RMET is considered an advanced test of ToM, as it assesses how accurately one can recognize emotions or mental states in facial expressions, which is a central construct of social cognition.

### Statistical Analysis

Statistical analyses were performed using Statistical Package for Social Sciences (SPSS) 20.0 (IBM Corporation, Armonk, NY, United States). Normal distribution was tested separately for the patient and control group with Kolmogorov–Smirnov tests for all variables. Even after logarithmic transformation most of the parameters were not normally distributed. Hence, we relied on non-parametric tests (Mann–Whitney *U* test). For each comparison the non-parametric effect size *r*_contrast_ was calculated with the formula $\text{r}=\frac{\text{Z}}{\sqrt{\text{N}}}$ (Rosenthal and DiMatteo, 2001). Here, *Z* is the standardized test statistic. Results are reported as mean ± standard deviation, if not stated otherwise. Significance levels were set to *p* < 0.05 with Bonferroni adjustments by taking the respective number of neuropsychological tests or behavioral questionnaires into account (accordingly adjusted *p* < 0.008 with six neuropsychological tests, and *p* < 0.006 with eight behavioral questionnaires). Effect sizes were interpreted as small (0.1 ≤ *r* ≤ 0.3), intermediate (0.3 < *r* ≤ 0.5) or strong (0.5 > *r*) according to Cohen (1988).

To explore which test parameters differentiate best between bvFTD and control subjects, non-parametric ROC curves were calculated for each test. ROC analysis is commonly used to quantify how accurate medical diagnostic tests discriminate between diseased and non-diseased subjects. The ROC curve as a graphical plot illustrates the performance of a binary classifier system when its discrimination threshold is varied. It shows the trade-off between true positive rate (sensitivity) and false positive rate (1-specificity) (Metz, 1978, 1986). The AUC was calculated as an effective and combined measure of sensitivity and specificity determining a test’s ability to discriminate between diseased and healthy populations. Here, an AUC of 1 represents a test with perfect discrimination, while an AUC of 0.5 is interpreted as useless (Hanley and McNeil, 1982).

Due to missing values in neuropsychological and behavioral parameters in our dataset (see **Table 2**), a missing data analysis was performed as missing data might complicate the interpretation of results (Ware et al., 2012). This analysis was conducted with the Missing Value Analysis procedure of SPSS 20.0, IBM Statistics. It revealed that most values were missing in self-report behavioral questionnaires in the bvFTD group, and the TMT. Datasets of some specific study centers showed similar missing patterns. In the further analyses, the technique of pairwise deletion was used to deal with missing values and to include all data available for each case.

## Results

### Clinical and Epidemiological Characteristics

There were no differences between bvFTD and control subjects for age (63.9 ± 9.6 vs. 66.1 ± 10.1 years; Student’s *t*-test *t*(127) = -1.18, *p* = 0.24), gender (female/male 38/48 vs. 22/21; χ^2^-test χ^2^(1, *N* = 129) = 0.56, *p* = 0.45), and education (3.6 ± 1.2 vs. 3.8 ± 1.3; Mann–Whitney *U* test *U* = 1632.5, *p* = 0.26). The patient group included 37 (43%) possible bvFTD and 49 (57%) probable bvFTD patients according to Rascovsky et al. (2011). In the following we will report data for the whole bvFTD group yielding sufficient statistical power. The FTLD-CDR score for the bvFTD group was 8.4 ± 5.4 and for the control subjects 0.1 ± 0.3, the CDR score averaged 6.2 ± 4.3 and 0.1 ± 0.2 (*U* = 5.5, 8.0, *p* < 0.001).

### Neuropsychological Tests – Group Comparisons

Performance in neuropsychological tests is shown in **Table 1**. Concerning executive functions and social cognition, the bvFTD group showed a significant decline as compared to healthy controls in every test, also after Bonferroni correction. Effect sizes were strong for fluency tests (phonemic/semantic fluency *r* = -0.72/-0.69), followed by the modified RMET (-0.64) and H5PT (-0.50), whereas intermediate effect sizes emerged for the Stroop task and TMT (-0.35 for both).

**Table 1:** Statistics for neuropsychological tests and behavioral questionnaires – Comparison between bvFTD and healthy control subjects.

	bvFTD patients		Healthy controls					
	Median	Mean (*SD*)	Median	Mean (*SD*)	*U*	*Z*	*p*	*r*
**Neuropsychological tests**								
Stroop task	96.7	86.18 (22.69)	100	98.87 (2.91)	552	-3.378	0.001^∗^	-0.35
Trail Making Test (TMT)	2.79	2.92 (1.16)	2.11	2.13 (0.45)	609.5	-3.391	0.001^∗^	-0.35
Hamasch Five-Point Test (H5PT)	77	67.31 (27.53)	95	93.65 (6.02)	414.5	-5.007	<0.001^∗^	-0.50
Phonemic fluency	7	7.41 (4.90)	18	17.83 (4.55)	192.5	-7.776	<0.001^∗^	-0.72
Semantic fluency	11	12.46 (6.71)	26.5	26.48 (4.72)	171	-8.079	<0.001^∗^	-0.69
Reading the Mind in the Eyes Test (RMET)	11	10.83 (3.79)	16	16.71 (2.56)	214.5	-6.272	<0.001^∗^	-0.64
**Behavioral questionnaires**								
Apathy Evaluation Scale (AES), self-report	21	20.26 (9.64)	9	9.79 (4.91)	91.0	-3.696	<0.001^∗^	-0.55
Apathy Evaluation Scale, informant-report	36	34.95 (10.00)	9	11.73 (6.63)	42.5	-5.504	<0.001^∗^	-0.61
Bayer Activities of Daily Living Scale (BADL), self-report	1.88	3.03 (2.66)	1.38	1.46 (0.46)	158.0	-2.933	0.003^∗^	-0.42
Bayer Activities of Daily Living Scale, informant-report	5.68	5.68 (2.79)	1.26	1.40 (0.50)	40.5	-5.395	<0.001^∗^	-0.59
Frontal Systems Behavioral Scale (FrSBe), frequency, self-report	45	51.04 (20.81)	36	37.00 (8.47)	153.5	-2.777	0.005^∗^	-0.40
Frontal Systems Behavioral Scale, frequency, informant-report	72	73.10 (17.97)	31	34.80 (9.35)	27.0	-5.650	<0.001^∗^	-0.64
Frontal Systems Behavioral Scale, distress, self-report	36	39.18 (14.09)	28	31.82 (10.14)	158	-1.989	0.047	-0.30
Frontal Systems Behavioral Scale, distress, informant-report	58.5	60.47 (17.15)	24	27.46 (6.00)	16.5	-5.365	<0.001^∗^	-0.64

*Significant with Bonferroni adjusted alpha level of ^∗^*p* < 0.008 for neuropsychological tests, *p* < 0.006 for behavioral questionnaires; Abbreviations: r, effect size; SD, standard deviation; U, Mann–Whitney U; *Z*, test statistic of Mann–Whitney U.*

### Behavioral Questionnaires – Group Comparisons

**Table 1** summarizes results for the behavioral questionnaires. In almost all behavioral questionnaires bvFTD patients showed a significant dysfunctional change in behavioral patterns as compared to healthy controls. Only the group difference for the degree of distress caused by dysfunctional behaviors as measured with the FrSBe self-report was not significant after Bonferroni correction.

Effect size analyses revealed higher values for informant- than self-report for frequency (*r* = -0.64/-0.40) and distress (-0.64/-0.30) caused by dysfunctional behaviors as measured with the FrSBe. Effect sizes of the FrSBe were strong for the informant-report and intermediate for the self-report. An analog pattern was found for the AES evaluating apathy. Here, informant-report (-0.61) indicated higher impairment than self-report (-0.55), although for both measures effect size was strong for discrimination between bvFTD and control subjects. Remarkably, for the BADL evaluating difficulties in activities of daily life the informant-report reached strong (-0.59) and the self-report reached intermediate effect size (-0.42), without differences between both measures.

### Discriminating between Groups – Receiver Operator Characteristic (ROC) Curves

**FIGURE 1:**
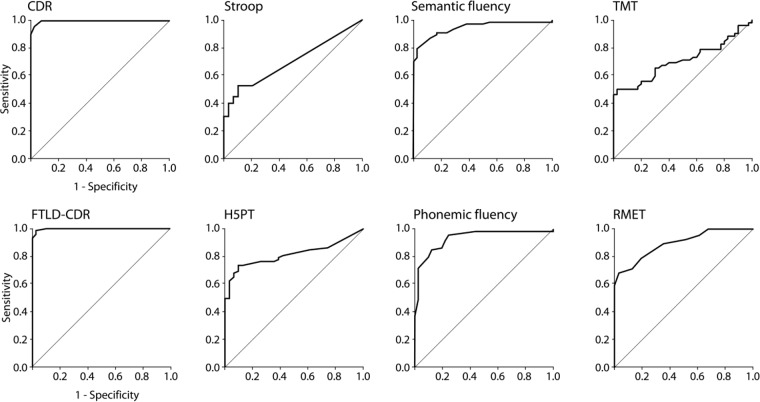
Receiver Operating Characteristic curves for the CDR, the CDR specified for FTLD (FTLD-CDR), and for the several neuropsychological tests, namely the Stroop test, H5PT, semantic and phonemic fluency, TMT, and the RMET.

**FIGURE 2:**
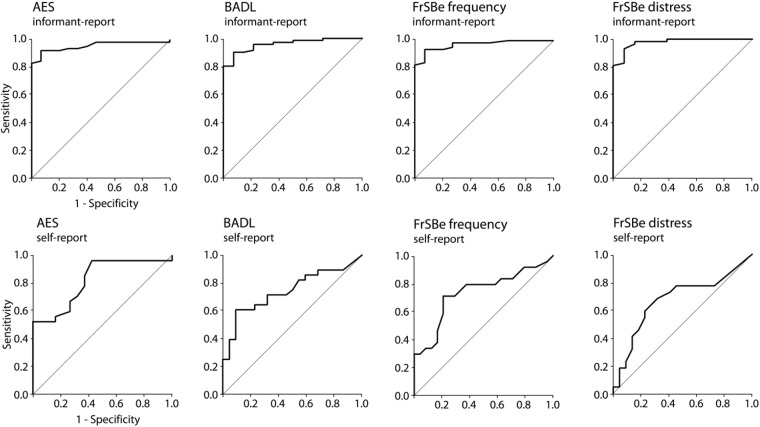
Receiver Operating Characteristic curves for the behavioral questionnaires, in particular the AES, BADL, and the FrSBe. Informant- and self-report measures are shown.

To explore how well the single neuropsychological tests or behavioral questionnaires discriminate between bvFTD and controls, we computed a non-parametric ROC curve for each parameter as illustrated in Figures 1, 2. The CDR and FTLD-CDR discriminated almost perfectly between bvFTD patients and healthy controls (**Figure 1**). The Stroop task, TMT, and H5PT had a higher specificity than sensitivity. Phonemic and semantic fluency tasks as well as the modified RMET on the other hand showed relatively balanced sensitivity and specificity. **Figure 2** illustrates that the AES in the self-report had a greater sensitivity than specificity. The graphical illustrations of the other behavioral questionnaires (AES in informant-report, BADL in both versions, and FrSBe in both versions) showed relatively balanced ratios of sensitivity and specificity.

**FIGURE 3:**
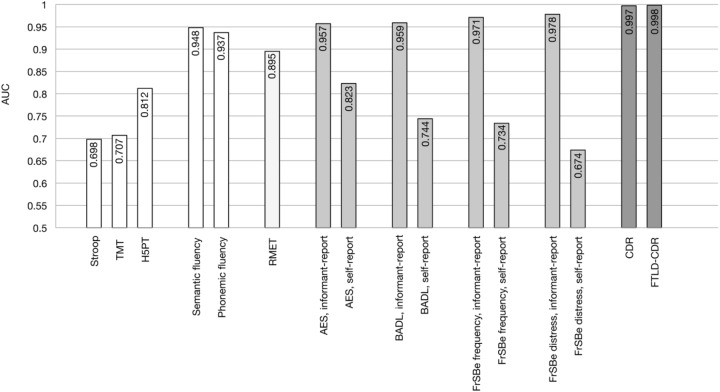
Discriminatory power as measured with Areas Under the Curve (AUC) as revealed by ROC curves for the AES, BADL, CDR, CDR specified for FTLD (FTLD-CDR), FrSBe, H5PT, RMET, and TMT.

To investigate the discriminatory power in distinguishing between bvFTD and control subjects the AUC was calculated for each single test. Results are shown in **Table 2** and **Figure 3**. Discriminatory power was highest for CDR and FTLD-CDR, and for the informant-report behavioral questionnaires, whereas the self-report questionnaires reached much lower values. The discrepancy between informant- and self-report was highest for FrSBe and BADL, and lower for AES. For neuropsychological tests, semantic and phonemic fluency reached highest values for discriminatory power, closely followed by the modified RMET, whereas the H5PT, TMT and Stroop task showed lowest discriminatory power as reflected in the AUC values.

**Table 2:** Statistics for neuropsychological tests and behavioral questionnaires in discriminatory power between bvFTD and healthy control subjects – Results of the AUC analyses.

				Available values (%)	
	Area Under Curve (AUC)	Confidence Interval	*p*	bvFTD	Controls
**Dementia severity measures**					
Clinical Dementia Rating Scale (CDR)	0.997	0.992–1.000	<0.001	84.9	93
Frontotemporal Lobar Degeneration (FTLD-)-CDR	0.998	0.994–1.000	<0.001	84.9	93
**Neuropsychological tests**					
Stroop task	0.698	0.593–0.803	0.002	73.3	67.4
Trail Making Test (TMT)	0.707	0.601–0.813	0.001	60.5	93
Hamasch Five-Point Test (H5PT)	0.812	0.730–0.893	<0.001	82.6	72.1
Phonemic fluency	0.937	0.893–982	<0.001	87.2	95.3
Semantic fluency	0.948	0.910–0.985	<0.001	90.7	97.7
Reading the Mind in the Eyes Test (RMET)	0.895	0.835–0.955	<0.001	76.7	72.1
**Behavioral Questionnaires**					
Apathy Evaluation Scale (AES), self-report	0.823	0.701–0.944	<0.001	31.4	44.2
Apathy Evaluation Scale, informant-report	0.957	0.915–0.999	<0.001	76.7	34.9
Bayer Activities of Daily Living Scale (BADL), self-report	0.744	0.606–0.881	0.003	32.6	51.2
Bayer Activities of Daily Living Scale, informant-report	0.959	0.918–0.999	<0.001	81.4	32.6
Frontal Systems Behavioral Scale (FrSBe), frequency, self-report	0.734	0.587–0.880	0.006	27.9	55.8
Frontal Systems Behavioral Scale, frequency, informant-report	0.971	0.939–1.000	<0.001	73.3	34.9
Frontal Systems Behavioral Scale, distress, self-report	0.674	0.508–0.839	0.049	25.6	51.2
Frontal Systems Behavioral Scale, distress, informant-report	0.978	0.946–1.000	<0.001	67.4	30.2

## Discussion

Our study investigated changes in neuropsychological functions in bvFTD with a focus on executive functions and social cognition, which are the cognitive dimensions mainly impaired in this disease affecting essentially frontal lobes (Rascovsky et al., 2011; American Psychiatric Association, 2013; Schroeter et al., 2014). The study was conducted in a large multi-centric sample and applied the new and revised diagnostic criteria by Rascovsky et al. (2011). In particular, the study examined the question whether a modified RMET as a measure for social cognition might predict bvFTD equal to or even better than executive function tests. In the following we will discuss the study’s results.

### Executive Function Tests Are Good Predictors for bvFTD

Our study confirmed that bvFTD is related to a significant decline in executive functions as demonstrated for all executive tests (Stroop task, TMT, H5PT, semantic and phonemic fluency). Results confirm earlier studies showing that executive functions including planning, organization, judgement, problem solving and mental flexibility are highly impaired in bvFTD (Gregory and Hodges, 1996). Interestingly, the most frequent finding was a constant deficit in the ability to produce semantic and phonemic wordlists (Pachana et al., 1996; Galante et al., 1999; Gregory, 1999; Pasquier et al., 1999; Perry and Hodges, 2000).

In line with these studies, verbal fluency tests predicted bvFTD better than other gold standard tests for executive functions like the H5PT, Stroop task, or TMT in our study as demonstrated by respective AUC values. Obviously, several executive tests tap into different aspects of executive functions and not all of these aspects are equally relevant for everyday life (e.g., Rabbitt, 1997; Torralva et al., 2009; Schroeter et al., 2012). Verbal fluency tests are based on the ability of spontaneous divergent thinking involving working memory, speech ability, attention and memory, whereas the other executive function tests are related to the ability to draw abstract patterns (H5PT), to connect alternating numbers and letters (TMT) as a measure of divided attention or to inhibit an overlearned response (Stroop task) (Rabbitt, 1997; Haid et al., 2002; Schroeter et al., 2002, 2012; Torralva et al., 2009). However, one has to keep in mind that deficits in executive functions, although prominent in bvFTD, are by far not disease-specific as they occur in other neurodegenerative diseases such as Alzheimer’s disease too (Schroeter et al., 2012). Already, Gregory et al. (1999, 2002) and Hodges (2007) criticized that executive function tests may fail to detect the onset of cognitive impairment in bvFTD patients.

### Social Cognition Tests Seem to Be Better Predictors for bvFTD

Most interestingly, our results for the modified RMET as one measure for ToM/social cognition provide evidence for a significant decline in recognition of mental/emotional states in bvFTD. Indeed, the RMET was a better diagnostic predictor for the diagnosis of bvFTD than executive measures such as the Stroop task, TMT and H5PT. Obviously, the RMET is related, beside others, to verbal abilities, since it requires the differentiation between semantically similar adjectives describing the person’s mental state, indicated by the eyes. This might, at least partly, explain comparable discriminatory power between the modified RMET and verbal fluency tasks. Other executive tasks rely less on verbal content (Stroop, TMT, and H5PT). We checked this hypothesis with a multiple regression analysis using the modified RMET as dependent variable and executive function tests as independent variables. Indeed, this analysis identified semantic fluency as the only significant parameter (beta = 0.52, *T* = 3.1, *p* = 0.004).

Our results are in line with previous research showing that ToM is a good diagnostic predictor for bvFTD (Gregory et al., 2002; Torralva et al., 2009; Adenzato et al., 2010) in agreement with the assumption that the frontomedian cortex, related to ToM processing (Amodio and Frith, 2006), has been regarded as the neural ‘hot-spot’ of bvFTD (Schroeter et al., 2008, 2011, 2014; Schroeter and Neumann, 2011; Schroeter, 2012; Meyer et al., 2017). A recent meta-analysis by Henry et al. (2014) involving 800 subjects confirmed the central role of ToM by showing significantly higher and domain-specific impairments in ToM (and emotion recognition) in bvFTD in comparison with control subjects and Alzheimer’s disease. Another new comprehensive systematic meta-analysis across 30 clinical conditions including multiple psychiatric, neurological and developmental disorders has replicated specific and strongest social cognitive dysfunction in bvFTD (large effect size with Cohen’s d -1.79 for ToM and -1.81 for facial emotion recognition; Cotter et al., 2018). To place our results into this framework we calculated effect sizes for the group comparison bvFTD vs. control subjects of the modified RMET in our study similar to Cotter et al. (2018). Remarkably, analyses revealed similar effect sizes of Cohen’s *d* = - 1.82 and Hedges’ *g* = - 1.70^2^. Specifically the RMET has been shown to predict very early bvFTD (Pardini et al., 2013), and to discriminate better between bvFTD and healthy controls or Alzheimer’s disease than executive tests (Gregory et al., 2002; Torralva et al., 2007, 2009; Gleichgerrcht et al., 2010; Buhl et al., 2013). Although verbal fluency tests performed comparable to the modified RMET in our study, they are based on several cognitive functions/abilities (see above) making these measures less specific for executive functions than other executive function tests such as the H5PT, TMT or Stroop task. Moreover, executive functions tests are not disease-specific in contrast to the social cognition tests such as the RMET for the differential diagnosis across different forms of dementia (see above).

Based on our results and literature data one can conclude that social cognition tests, such as the RMET, are better and more disease-specific predictors for bvFTD than executive function tests. One might object that empathy is already contained in the diagnostic criteria for bvFTD making the inclusion of social cognition/ToM redundant (Rascovsky et al., 2011; American Psychiatric Association, 2013). However, ToM refers to the cognitive understanding of an emotional or mental state, while empathy refers to the emotional sharing of an emotional state (Hein and Singer, 2008; Bzdok et al., 2012). Neural correlates of empathy and ToM are regionally dissociated and overlap only partly (Bzdok et al., 2012; Schroeter et al., 2014). We suggest including a decline of social cognition/ToM in future revisions of the diagnostic criteria for bvFTD in the International Classification of Diseases (ICD) version 11 by the World Health Organization as already suggested by the DSM-5 to increase their specificity, reliability and predictive power (Schroeter, 2012).

### Behavioral Questionnaires Are Best Predictors for bvFTD

Finally, we want to discuss results for behavioral measures. The three questionnaires AES, BADL and FrSBe showed in both versions (self-report and informant-report) a significant increase of dysfunctional behavioral patterns and everyday life difficulties in bvFTD as compared to healthy controls. The informant-reports were consistently better diagnostic predictors for bvFTD than self-reports reflecting the lack of patient’s insight concerning their disease and emphasizing the importance of informant-reports for correct and early diagnosis of bvFTD.

**FIGURE 4:**
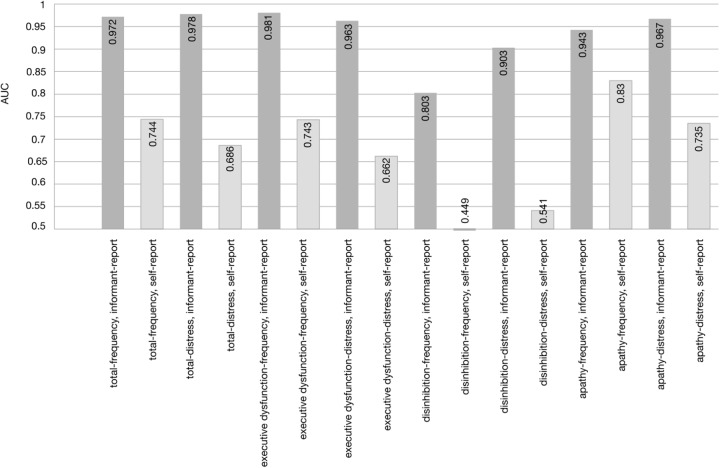
Discriminatory power as measured with Areas Under the Curve (AUC) as revealed by ROC curves for the total scale and the subscales of the FrSBe.

The informant-report of the FrSBe, which assesses typical behavioral patterns of the frontal system, including apathetic, disinhibited behavior, and executive functions in daily living, showed a higher diagnostic prediction of bvFTD than the AES. The latter test exclusively focuses on apathy. A similar advantage of the FrSBe emerged against the BADL, a scale taping into general difficulties in everyday activities. The higher prediction of bvFTD by the FrSBe can be explained by the fact that it assesses more than only one single dysfunctional behavioral pattern. Although less than the informant-reports, self-reports also indicated significantly more dysfunctional behavioral patterns in bvFTD than in healthy controls. The bvFTD patients did thus have at least some insight into the disease. Interestingly, apathy as assessed in the AES self-report questionnaire had the highest AUC in self-reports as compared to the BADL and the FrSBe, which might be related to a selective loss of insight. This pattern is also mirrored in an item-specific AUC analysis of the FrSBe scale, where patients’ self-report for apathy discriminated better than self-report for executive dysfunction or disinhibition (**Figure 4**).

Dysfunctional behavioral patterns as assessed by AES, BADL and FrSBe in the informant-report were better predictors of bvFTD than all other neuropsychological (executive and social cognitive) tests investigated. Although not surprising as diagnostic systems define bvFTD exactly by these behavioral impairments (Rascovsky et al., 2011; American Psychiatric Association, 2013), our results suggest that dysfunctional behavioral patterns shall be measured with quantitative instruments for a reliable and correct early diagnosis of bvFTD in clinical routine in addition to assessing neurocognitive decline with neuropsychological tests.

### Limitations of the Study and Perspectives

Several caveats should be kept in mind when considering the current results. Although the multi-centric study design might have confounded results, this bias was minimized by standard operating procedures (SOPs) and investigator training. We applied a shortened and modified 24 item version of the RMET containing 18 original RMET stimuli and six basic emotion stimuli. We chose such a combined measure, because both, ToM and emotion recognition, are similarly impaired in bvFTD (Henry et al., 2014). We aimed at increasing sensitivity but might have lost test specificity. Subanalyses could not be performed due to low item numbers for tests. Applying a shortened version of the RMET might hamper the detection of mild or sub-threshold impairments in social cognition. Accordingly, the 36-item full version of the RMET might be given preference in the future, especially for mild and pre-stages such as mild behavioral impairment (Poletti et al., 2013). Future studies shall also disentangle impairments in ToM and emotion recognition or cognitive and affective components of ToM, by applying several tests, because social cognition/ToM are complex constructs and contain several aspects (Frith and Frith, 2003; Amodio and Frith, 2006; Poletti et al., 2012; Enrici et al., 2015). For executive function tests, future studies shall include additionally tests investigating executive abilities in daily living settings such as the Behavioral Assessment of the Dysexecutive Syndrome (BADS) test battery (Schroeter et al., 2012).

Our study focused on two cognitive dimensions, executive functions and social cognition without exploring other cognitive abilities, because these cognitive dimensions have been suggested as mainly impaired in bvFTD in the literature (Torralva et al., 2009), and to focus the paper on the most important research question. Analyzing additionally all other cognitive dimensions (attention, memory functions, language) might be a desideratum for future studies to strengthen our hypothesis.

Our study discriminated patients with bvFTD very well from healthy controls. One might ask whether such analyses are also suited to separate patients with possible and probable bvFTD. To answer this question the same analyses were conducted for this comparison. Remarkably, no group differences were detected between possible and probable bvFTD for all neuropsychological tests and behavioral questionnaires if the same analysis criteria were applied (in particular Bonferroni correction). The only parameter that could separate both bvFTD subgroups in the ROC analyses was phonemic fluency with an AUC of 0.639 and *p* = 0.039 (mean values for possible/probable bvFTD 8.64 ± 4.91, 6.45 ± 4.73).

A further critical aspect in the general investigation of bvFTD is that these patients may have a reduced willingness to participate in a study due to their dysfunctional behavioral pattern and loss of insight. We assume that a high number of missing data might actually be due to dysfunctional behavior in bvFTD patients, in particular in self-report in behavioral questionnaires. While bvFTD cases had more missing values in self-reports, healthy controls had more missing values in the informant-reports, most probably because they had to return the questionnaires per mail and that they might not have understood the relevance of their contribution to the study. Another frequent missing pattern showed TMT missing values in bvFTD patients, although all other tests were complete. We assume that in these patients that obviously were compliant, the TMT was either not assessed or at least stopped due to a more severe cognitive decline. Furthermore, the data from some study centers offered a high percentage of identical missing patterns which suggests a methodological center bias here. Due to missing values we had to perform separate ROC analyses instead of a complete case ROC analysis which might be regarded as a statistical limitation. Although the resulting analyses were consequently based on slightly differing group constitutions, we assume, however, that our subsamples were rather homogeneous and allow comparisons between them.

## Conclusion

Our study aimed at identifying those neuropsychological and behavioral parameters with best discriminating power between bvFTD, defined by new diagnostic criteria, and age-matched healthy controls in a large multi-centric sample. The study focussed on executive dysfunction and impairments in social cognition as most characteristic features of bvFTD. Patients performed significantly worse than healthy controls in all neuropsychological tests. Discriminatory power (AUC) was highest in behavioral questionnaires, followed by verbal fluency tasks, the social-cognitive RMET and executive function tests such as the Stroop task, TMT and H5PT. As fluency tasks depend on several cognitive functions, not only executive functions, results suggest that the social cognition test – the applied modified RMET – discriminated better between bvFTD and controls than ‘proper’ executive tests. Our findings and data from other studies strengthen the argument that tests for social cognition, such as the RMET, shall be incorporated into standard clinical batteries and diagnostic criteria in the future as already suggested in bvFTD’s DSM-5 criteria. The study underlines the diagnostic potential of neuropsychological assessments and behavioral questionnaires to confirm bvFTD diagnoses in clinical practice.

## Author Contributions

General conception: MS, AD, IU, MO, and JD-S. Study design: MS, SP, AD, IU, MO, and JD-S. Data analysis: SP. Figures: MS and SP. Drafting the manuscript: MS and SP. Final preparation of the article: MS, SP, SB, JaK, KS, MP, SA-S, AD, KF, HJ, FJ, JoK, ML, JP, AS, IU, AT-O, MO, and JD-S.

## Conflict of Interest Statement

The authors declare that the research was conducted in the absence of any commercial or financial relationships that could be construed as a potential conflict of interest.

## Footnotes

1 http://www.ftld.de

2 http://www.socscistatistics.com/effectsize/Default3.aspx

## Abbreviations

AES: Apathy Evaluation Scale
AUC: area under the curve
BADL: Bayer Activities of Daily Living Scale
bvFTD: behavioral variant frontotemporal dementia
CDR: Clinical Dementia Rating Scale
DSM: Diagnostic and Statistical Manual of Mental Disorders
FrSBe: Frontal Systems Behavioral Scale
FTLD: frontotemporal lobar degeneration
FTLD-CDR: FTLD modified CDR
H5PT: Hamasch-Five-Point Test
ISCED: International Standard Classification of Education
RMET: Reading the Mind in the Eyes Test
ROC: receiver operating characteristic
TMT: Trail Making Test
ToM: Theory of Mind
